# YOLO-ISTD: An infrared small target detection method based on YOLOv5-S

**DOI:** 10.1371/journal.pone.0303451

**Published:** 2024-06-13

**Authors:** Ziqiang Hao, Zhuohao Wang, Xiaoyu Xu, Zheng Jiang, Zhicheng Sun

**Affiliations:** National Demonstration Center for Experimental Electrical, School of Electronic and Information Engineering, Changchun University of Science and Technology, Changchun, China; VIT-AP Campus, INDIA

## Abstract

Infrared target detection is widely used in industrial fields, such as environmental monitoring, automatic driving, etc., and the detection of weak targets is one of the most challenging research topics in this field. Due to the small size of these targets, limited information and less surrounding contextual information, it increases the difficulty of target detection and recognition. To address these issues, this paper proposes YOLO-ISTD, an improved method for infrared small target detection based on the YOLOv5-S framework. Firstly, we propose a feature extraction module called SACSP, which incorporates the Shuffle Attention mechanism and makes certain adjustments to the CSP structure, enhancing the feature extraction capability and improving the performance of the detector. Secondly, we introduce a feature fusion module called NL-SPPF. By introducing an NL-Block, the network is able to capture richer long-range features, better capturing the correlation between background information and targets, thereby enhancing the detection capability for small targets. Lastly, we propose a modified K-means clustering algorithm based on Distance-IoU (DIoU), called K-means_DIOU, to improve the accuracy of clustering and generate anchors suitable for the task. Additionally, modifications are made to the detection heads in YOLOv5-S. The original 8, 16, and 32 times downsampling detection heads are replaced with 4, 8, and 16 times downsampling detection heads, capturing more informative coarse-grained features. This enables better understanding of the overall characteristics and structure of the targets, resulting in improved representation and localization of small targets. Experimental results demonstrate significant achievements of YOLO-ISTD on the NUST-SIRST dataset, with an improvement of 8.568% in mAP@0.5 and 8.618% in mAP@0.95. Compared to the comparative models, the proposed approach effectively addresses issues of missed detections and false alarms in the detection results, leading to substantial improvements in precision, recall, and model convergence speed.

## Introduction

With the rapid development of computer vision and deep learning, target detection technology has been widely used in various fields. Infrared target detection has been widely used in industrial fields, including environmental monitoring and autonomous driving, due to its ability to accurately detect temperature differences between different objects.

Currently, infrared small target detection can be divided into two main directions: traditional target detection methods and current advanced deep learning methods. Both traditional and deep learning methods were originally developed for visible light images, and although they have achieved remarkable results in the field of visible light images, there are certain challenges in applying these algorithms directly to the task of infrared small target detection [1].

Traditional methods typically use hand-crafted feature extraction algorithms that are often insensitive to the complex texture, shape and scale variations of small infrared targets, resulting in limited detection performance. In addition, since there is often a large amount of background clutter information in infrared images, it is difficult for traditional methods to effectively suppress the background noise, leading to interference in the detection results and a high false alarm rate. Therefore, traditional methods often fail to achieve better detection results in complex infrared scenes.

Deep learning is a technique based on the learning of surface features. Through multiple layers of surface feature learning, high-level feature representations of raw data are progressively obtained, eliminating the need for manual feature input and extraction. [2] exhibits remarkable advantages compared to traditional techniques, such as automatic feature extraction, impressive efficiency, simplicity, and higher accuracy. These networks, by processing large datasets, possess the ability to discern and learn complex features, thereby enhancing target recognition capabilities [3].

In contrast, deep learning methods are able to effectively capture target details and contextual information through the learning and feature extraction capabilities of deep neural networks. However, the direct application of deep learning methods to infrared small target detection still faces some problems:

1. The low resolution and small target size of infrared images make it difficult to clearly distinguish and accurately locate the target in the image.
2. Phenomena such as background interference and target occlusion are often present in infrared images, further increasing the challenge of target detection.
3. It is difficult to distinguish small target features from noise because the deep-shallow and global-local feature association is not established.
4. Multiple downsampling leads to the loss of information about small targets in the deep feature map, which seriously reduces the detection capability of the model.

To address the above issues, this paper is dedicated to the improvement of Yolov5-S to enhance its performance in infrared small target detection tasks. The main contributions of this paper are as follows:

- To address the low resolution and small target size in infrared images, we have designed a novel K-means algorithm based on the DIOU (Distance-IoU) metric. This algorithm is specifically tailored to better adapt to the small target sizes found in infrared images, thereby improving the accuracy and stability of target detection.
- To address the challenges of small target scales and the loss of detail information caused by multiple downsampling in infrared images, we propose a feature extraction module called SACSP. This module is designed to reconstruct the backbone of the YOLOv5-S detector, avoiding the blurring of details in small targets and preserving their key information, thus improving the performance of the detector. Additionally, we modify the detection heads in YOLO by replacing the original 8, 16, and 32 times downsampling detection heads with 4, 8, and 16 times downsampling detection heads.
- To address the problem of low discrimination between targets and backgrounds in infrared images, we modified the original SPPF module, and by introducing NL-Block, we were able to introduce global features in the SPPF module, that better capture the association between background information and the target, enabling the network to better localise the target and improve the detection capability of small targets.

## Related work

### Infrared target detection

Current infrared target detection methods can be broadly divided into two categories. One class is the traditional detection methods based on artificially designed features, including background feature-based methods [4–8], which use the differences between the target and the background for detection, for example by modelling and analysing the statistical properties of the background to detect the target, target feature-based methods [9–15], which focus on extracting the features of the target itself, such as shape, texture, colour, etc., and then using these features for target detection, In addition, methods based on low-rank and sparse decomposition [16–20] are often applied to infrared target detection, which exploit the low-rank and sparse nature of the target in infrared images.

The other category is deep learning based methods, there are improvements based on existing excellent target detection models (e.g. Faster RCNN [21], SSD [22], YOLO [23], etc.) [24, 25], which have already achieved good detection performance in visible light images, and can be extended to the infrared target detection task by making appropriate adjustments and optimisations to infrared images. In addition, there are also some researchers who perform the detection of small infrared targets using their own designed models [26–29], which usually take into account the special characteristics and requirements of infrared images to improve the detection performance.

### YOLO network

In 2016, Redmon et al. [23] proposed an end-to-end target detection network called YOLO (You Only Look Once), an approach that has received widespread attention. By integrating the target detection process into a single neural network, YOLO was able to significantly reduce the time required for target detection and performed well in terms of accuracy. With the proposal of YOLOv1, single-stage target detection methods gradually received more attention. Subsequently, several updates of the YOLO network have been released, from 1.0 to 8.0 [30–34]. The continuous development of these versions integrates the factors of detection accuracy, detection speed and network size, among which the YOLOv5 network is more representative and popular. In the YOLOv5 network, the CSPDarknet53 is used as the backbone network and the Path Aggregation Network (PAN) is introduced as the feature fusion module. By using optimisation modules (e.g. SPPF) and data enhancement modules (e.g. Mosaic Enhancement), the detection performance of the YOLOv5 network has been greatly improved. In addition, the network introduces a scale factor design that allows the YOLOv5 network to be adapted to multiple networks of different sizes for different application scenarios, further improving its applicability. Researchers have improved and extended YOLOv5 to design some domain-specific target detection networks. Among them, Li R et al [35] proposed YOLOSR-IST, a deep learning method for small target detection in infrared remote sensing images based on super-resolution, and YOLO, which combines the ideas of YOLOv5 and Swin Transformer. Mou et al [36] designed YOLO-FR, a network focusing on infrared small target detection. These works enrich the application scenarios of the YOLO family of networks and provide more options for domain-specific target detection tasks.

### Feature fusion

In deep learning, features at different levels carry information about the original image at different scales, high-level features contain the position of smaller objects and low-dimensional feature information, while low-level features contain the position of larger objects and high-dimensional feature information. The role of feature fusion is to extract and express richer and more discriminative features, thus improving the performance and generalisation of the model. Zhang et al [37] proposed the CHFNet, a novel infrared target detection method that uses a locally fused HLF module as a cross-layer feature fusion module, which significantly reduces the loss of feature information and improves the detection performance of weak infrared targets. Dai et al [38] proposed an infrared small target detection method that introduces an asymmetric contextual modulation module (ACMM) to extract target features more efficiently. Zhang et al [39] proposed ISNet and designed Taylor Finite Difference (TFD)-inspired edge blocks and Two-Way Attention Aggregation (TOAA) blocks to efficiently extract weak infrared targets by exploiting edge features in the ambiguous background. Yao et al [40] introduced the FCOS model, which suppresses the background noise by improved spatial feature fusion and traditional filtering methods to improve small target detection. Li et al [41] proposed the YOLO-FIRI model, which improves the structure of the feature extraction network and improves the accuracy of infrared small target detection by using a multi-scale structure.

## Methods

### Overview of YOLO-ISTD

**Fig 1:**
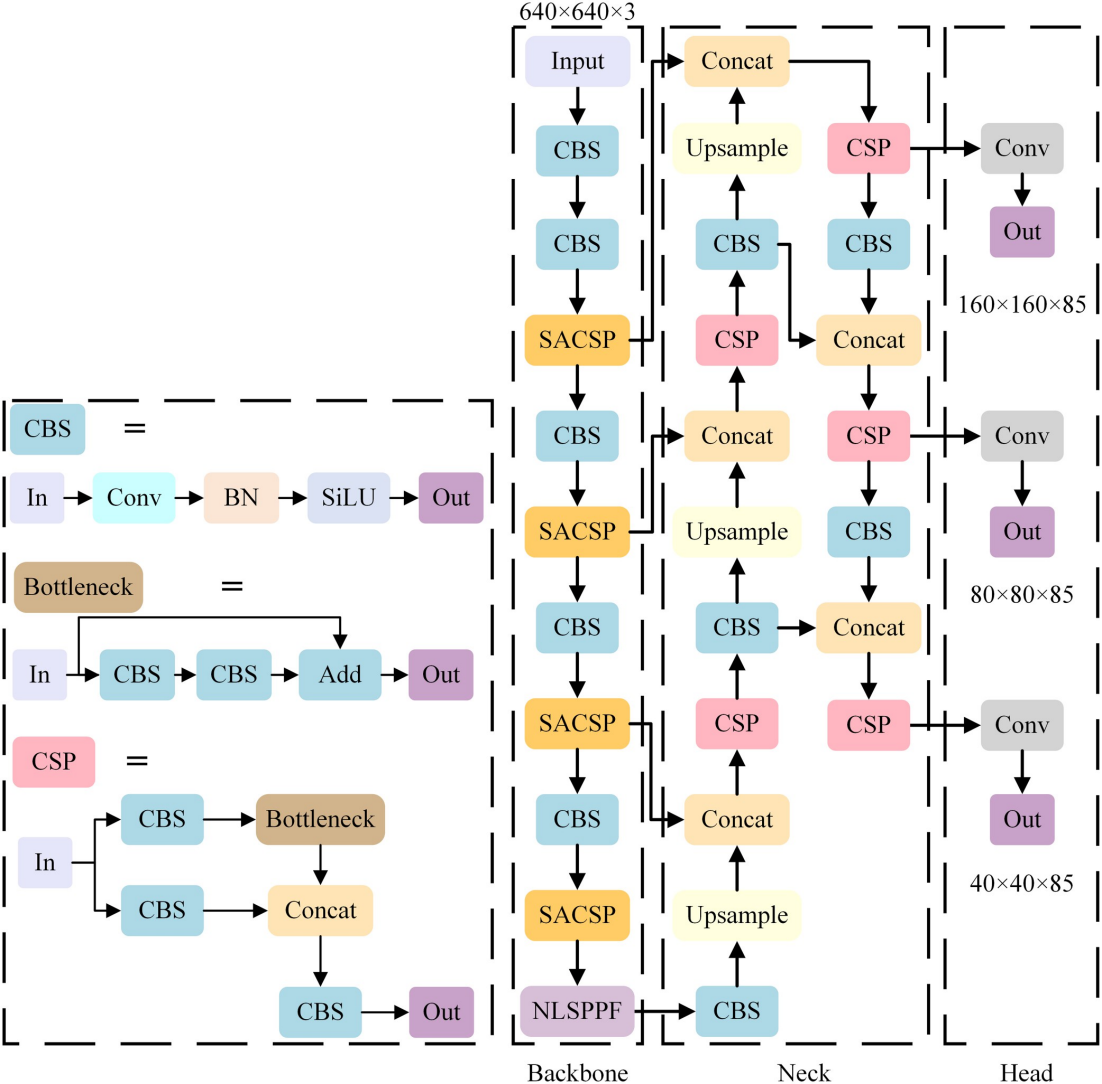
Structure of YOLO-ISTD.

The structure of the enhanced YOLO-ISTD detection model is shown in Fig 1, and this structure has been carefully optimised to detect small IR targets more effectively.

After extracting features from the backbone network, the model generates four feature maps of different sizes, each capturing information at different scales. These feature maps pass through the Neck feature fusion module. The deepest feature maps pass through the NLSPPF module of this paper before entering Neck for subsequent feature fusion.

We have modified the CSP module in Backbone to improve the feature extraction capability. In addition, we modified the detector head in YOLO by replacing the original 8-, 16-, and 32-fold downsampling detector heads with 4-, 8-, and 16-fold downsampling detector heads.

### SACSP module

**Fig 2:**
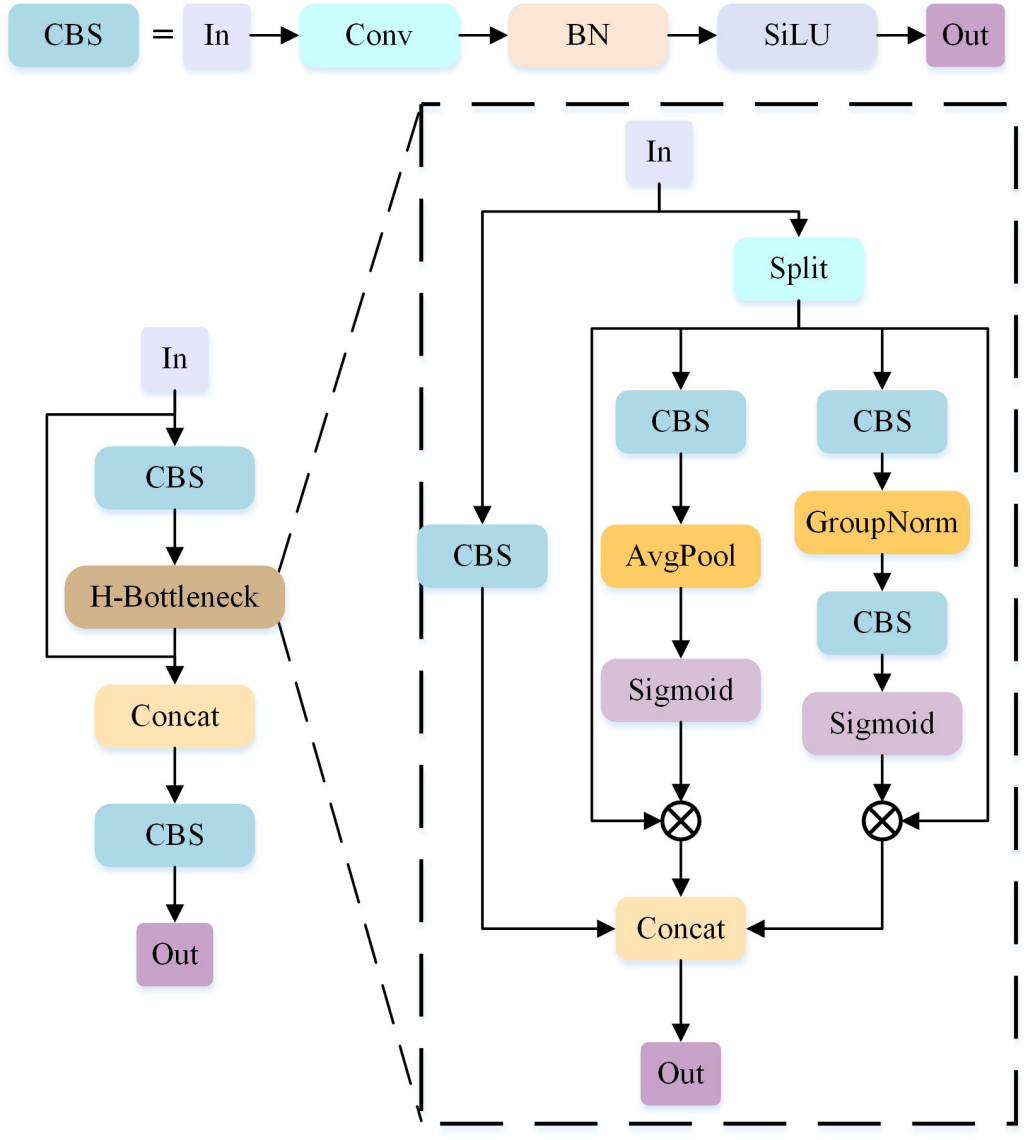
SACSP structure.

In the YOLOv5-S algorithm, the CSP module serves the purpose of feature extraction, extracting and learning high-level semantic features of images from the input feature map. However, the CSP module is designed based on the characteristics of large visible-light objects, which led us to analyze its limitations, especially regarding the decreased detection performance for small infrared targets. We discovered that the CSP module may not fully capture the detailed information of small infrared targets, leading to a decrease in detection accuracy. Additionally, due to the multiple downsampling operations within the CSP module, the contextual information of smaller targets may be lost, further impacting the detection results. The Bottleneck in the CSP module performs a 1 × 1 convolution that reduces the number of channels. However, the 1x1 convolution operation cannot capture a larger receptive field, which may result in the loss or reduction of information when the channel number of the input feature map is significantly compressed. This can have a negative impact on feature representation and model performance. In order to improve the performance of infrared small target detection, we try to adapt the structure of the CSP module by incorporating the shuffle attention mechanism and redesigning an improved CSP module called SACSP to provide better contextual information, as shown in Fig 2 for the structure of the SACSP module.

The SACSP module will retain the original residual structure of the CSP module during the improvement process, because the residual structure can transfer information across layer connections, which allows the network to better utilize the association between the bottom and top features. Therefore, in the improvement process, this paper will focus on improving the feature extraction part of the CSP module to further enhance the overall network performance.

We have removed the 1x1 convolution operation that reduces the number of channels in the CSP module, while retaining the important 3 × 3 downsampling operation. Additionally, we have replaced the Bottleneck part with the H-Bottleneck. The H-Bottleneck introduces the Shuffle Attention attention mechanism. By rearranging the channels and introducing attention weights into the feature map, we achieve effective information interaction at the channel level. This channel-level attention mechanism gives more weight to the important channels, which enhances the expressive power of the feature representation and improves the discriminative ability and generalization performance of the model.

The retention of the 3 × 3 downsampling operation aims to obtain deep-level features after downsampling, and the feature map after passing through the Shuffle Attention attention mechanism is fused with the feature map after downsampling through the Concat operation, thus introducing richer contextual information and guiding the model to understand the shallow, local, small-target detail features through the deep global semantic features. By capturing features at different scales, the model can better understand the structures and relationships in the image. Second, this operation can help the model learn richer and more diverse feature representations. By fusing downsampled features at different levels, the model can synthesise information from different levels to obtain a more expressive feature representation.

### NLSPPF module

In the YOLOv5-S algorithm, the role of the SPPF module is to fuse different levels of features to extract multi-scale features. However, due to the presence of pyramid pooling operation, the SPPF module may lead to the loss of some spatial information, which may affect the accurate localization ability especially when dealing with small-size targets or target boundaries. In addition, since the background and environment in infrared images have a large impact on target recognition, the traditional SPPF structure only fuses features by cascading pooling results at different scales, which may lead to the problem of information loss.

**Fig 3:**
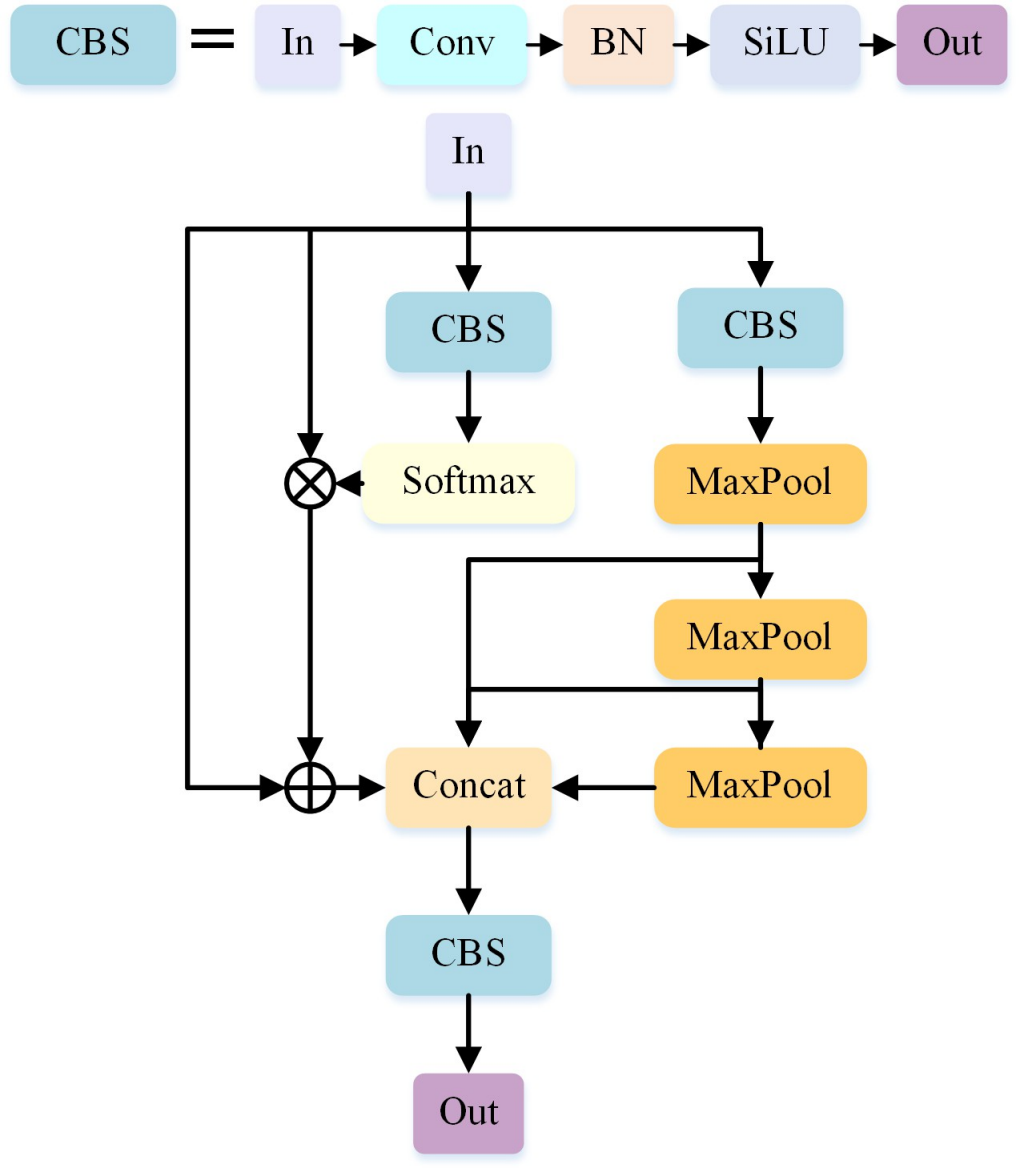
NLSPPF structure.

In order to solve the above problems, we designed an improved SPPF structure called NLSPPF, as shown in Fig 3. In NLSPPF, We retained the three successive MaxPool operations in the SPPF module because they gradually reduce the spatial size of the feature map while preserving important features. Each MaxPool layer performs spatial downsampling on the feature map, allowing the network to capture features at different scales, ranging from global to local. By applying multiple MaxPool operations, the model can progressively learn feature representations that are more translation-invariant. Additionally,we have introduced an NL-Block as an additional component. The NL-Block module enhances the expressiveness of small-size target feature representations by introducing non-local operations to compute the association between the background and the target features in the global range in the feature map. It can better understand the overall structure and semantic relationships in the image and provide more discriminative feature representations for further localization of small-size target locations and edges, thus improving the detection performance. Lastly, we have removed the Concat operation that performs channel reduction with a 1x1 convolution on the input, and instead, we include the output of the NL-Block in the Concat operation. The channel reduction with a 1x1 convolution can introduce redundant feature information, especially during feature fusion and concatenation. By removing this operation, the model can focus more on learning the crucial features from the input, reducing the interference of redundant information. By adding the NL-Block module to the Concat part, we can better model long-range dependencies between different positions in the image and capture richer global contextual information.

These improvements enable NLSPPF to handle small-sized targets and address the challenge of small target detection in complex infrared images more effectively. It overcomes the issue of information loss that exists in traditional SPPF structures.

### Modification of preset anchor with improved K-means

YOLOv5’s default anchor is obtained by clustering on the COCO dataset, which is designed to adapt to general target detection tasks. While the targets in the COCO dataset are mainly common objects in visible images, there are differences in physical properties between infrared and visible images, and small targets in infrared images tend to have lower pixel intensities and smaller sizes, so the default anchor size may not be optimal for detecting small infrared targets.

We developed an improved K-means algorithm based on DIoU (Distance-IoU) to obtain a suitable anchor. DIoU is a distance metric that can measure the distance between target frames more accurately than the traditional IoU (Intersection over Union) algorithm. By introducing DIoU into the K-means clustering algorithm, we are able to more accurately match the range of scales and aspect ratios of small infrared targets.

In the original K-means clustering algorithm, SSE is used to represent the effectiveness of clustering, where a smaller SSE value indicates a better result.

$$\begin{array}{c} SSE\left(C\right)=\sum_{k=1}^{n}\;\sum_{x_{i}\in C_{k}}{\left|x_{i}-m_{k}\right|}^{2} \end{array}$$ (1)

Among them, C is the collection of data after clustering, defined as C = *C*_1_, *C*_2_, …, *C*_*n*_; *m*_*k*_ is the centroid of cluster *C*_*k*_, and it is calculated as follows:

$$\begin{array}{c} m_{k}=\frac{\sum_{x_{i}\in C_{k}}x_{i}}{|C_{k}|} \end{array}$$ (2)

As you can see, the calculation of SSE is essentially the computation of the squared Euclidean distance between each data point in a cluster and its corresponding centroid.

However, for target detection tasks, the Euclidean distance metric is not always suitable. This is because the Euclidean distance only considers the straight line distance between points, while in target detection, the positional offset of the target frames is also very important. Therefore, in order to measure the distance between target frames more accurately, we redesign the distance metric based on the idea of DIOU.

The improved distance metric formula is as follows:

$$\begin{array}{c} \bar{\text{SSE}(C)}=d_{2}+expit(\frac{S_{m}}{S_{T}})+(1-\frac{x_{i}\in M^{\left\{x_{1},\ldots ,x_{n},x_{T}\right\}}*\min_{\begin{array}{c} y_{i}\in M^{\left\{y_{1},\ldots ,y_{n},y_{T}\right\}} \end{array}}}{S_{m}+S_{T}-\min_{\begin{array}{c} x_{i}\in M^{\left\{x_{1},\ldots ,x_{n},x_{T}\right\}}* \end{array}}\min_{\begin{array}{c} y_{i}\in M^{\left\{y_{1},\ldots ,y_{n},y_{T}\right\}} \end{array}}}) \end{array}$$ (3)

Whereas, *d*_2_ represents the squared Euclidean distance between each data point in a cluster and its corresponding centroid, and it is calculated as follows:

$$\begin{array}{c} d_{2}=\sum_{\left({\bar{x}}_{k},{\bar{y}}_{k}\right)\in m_{k}}^{n}\sum_{\left(x_{i},y_{i}\right)\in C_{k}}{\left|\left(x_{i}-{\bar{x}}_{k}\right)+\left(y_{i}-{\bar{y}}_{k}\right)\right|}^{2} \end{array}$$ (4)

where *S*_*m*_ represents the sum of areas of the nine selected center cluster points. The specific calculation method is as follows:

$$\begin{array}{c} S_{m}=\sum_{\begin{array}{c} i=1\\ \left(x_{i},y_{i}\right)\in m_{i} \end{array}}^{n}\left(x_{i}*y_{i}\right) \end{array}$$ (5)

where *S*_*T*_ represents the sum of areas of the target boxes. The specific calculation method is as follows:

$$\begin{array}{c} S_{T}=x_{T}*y_{T} \end{array}$$ (6)

In the above equations, *x*_*T*_ represents the x-coordinate of the detected target box, and *y*_*T*_ represents the y-coordinate of the detected target box. C represents the collective data after clustering, defined as C = {*C*_1_, *C*_2_, …, *C*_*n*_}. *m*_*k*_ = (*x*_*k*_, *y*_*k*_) represents the centroid of cluster *C*_*k*_, and correspondingly, we have the set M = {*m*_1_, *m*_2_, …, *m*_*n*_}.

By using this improved distance metric formula, we are able to more accurately calculate the distance between target boxes, which in turn improves the accuracy of clustering and the ability to generate an Anchor suitable for the task.

**Fig 4:**
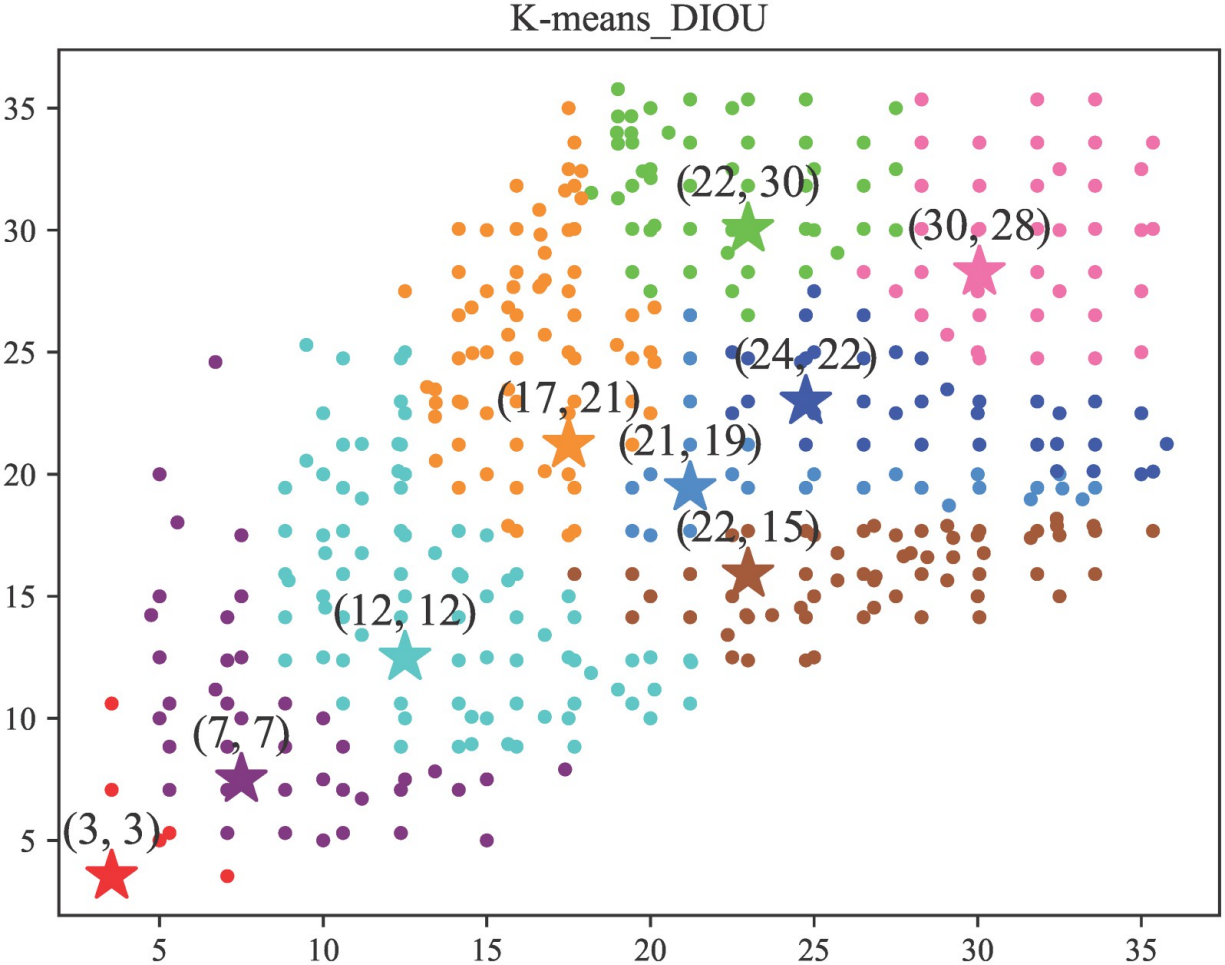
Effectiveness after clusteringe.

The final nine Anchors are [[3, 3], [7, 7], [12, 12], [17, 21], [21, 19], [22, 15], [22, 30], [30, 28], [24, 22]], and Fig 4 shows the effect of clustering.

### Detection head size modification

The input feature images of the three detection heads in YOLOv5-S are downsampled by 8, 16, and 32 times of the original image. Considering the small target scale of small infrared targets and the problem of insufficient detail information due to multiple downsampling, we modified the detection heads by changing the size of the input feature maps to downsampling by 4, 8, and 16 times.

The operation of doubling the size of the detection head usually increases the ability of the detector to sense small targets. When the target is small, the original detection head may not be able to capture detailed information about the target, resulting in missed detection or inaccurate localisation. By enlarging the size of the detection head, the resolution of the feature map can be increased, allowing small targets to be better represented and localised.

## Experiment and result

### Data set selection and training strategy

#### Data set selection

This study utilizes the NUST-SIRST infrared small target dataset to evaluate the performance of the proposed method. The NUST-SIRST dataset [42] contains 10,000 training images and 100 test images (image pixel sizes are around 128 × 128).

During training the dataset was randomly divided between training data and evaluation data in the ratio of train:val = 9:1.

#### Implementation details and training strategies

All training was conducted on a Linux operating system with an Intel(R) Xeon(R) Platinum 8255C CPU @ 2.50 GHz, GeForce RTX 3080, 10.0 GB of RAM, Pytorch version 1.13.0 and Cuda version 12.0.

No pre-training weights are loaded and training is performed directly on the NUST-SIRST dataset. In training, an Adam optimizer with a smoothing constant of 0.9 is used to optimize the MSE loss function. The learning rate for training is 10^−4^. The training iteration is 100 epoch, the number of working threads is 8, and the batch-size is 32.

### Comparative testing with state-of-the-art methods

The proposed method and several SOTA methods are tested on the NUST-SIRST dataset. Table 1 shows the comparison of the test results on the dataset.

**Table 1:** Comparison of results on the NUST-SIRST dataset.

Model name	precision	recall	F1	map@0.5	map@0.95	Params(M)
YOLOv5-S	81.814%	68.449%	73.605%	78.897%	38.344%	7.2
YOLOv5-L	82.591%	67.467%	73.939%	80.28%	41.171%	46.5
YOLO-FR	79.797%	72.973%	76.964%	83.671%	45.016%	4.8
YOLO-ViT	86.145%	70.33%	77.420%	83.061%	38.199%	6.2
YOLOv8-S	76.64%	73.236%	74.628%	80.939%	46.461%	11.2
Ours	81.923%	**82.561%**	**82.722%**	**87.465%**	**46.962%**	5.5

Based on the results from the NUST-SIRST experimental dataset, we can conclude that YOLO-ISTD performs better in detecting small infrared targets. Specifically, YOLO-ISTD achieved a score of 87.465% on the mAP@0.5 metric, which is the most comprehensive detection capability, outperforming other methods compared to it. Meanwhile, YOLO-ISTD’s mAP@0.95 also achieves the best score of all the methods compared. In addition, the recall of YOLO-ISTD is much higher than the other methods compared, reaching 82.561%. The F1 is also significantly higher than other comparative methods, reaching 82.722%. This means that YOLO-ISTD not only effectively reduces the probability of false alarms, but also significantly reduces the omission of real targets, which is common in other methods.

**Fig 5:**
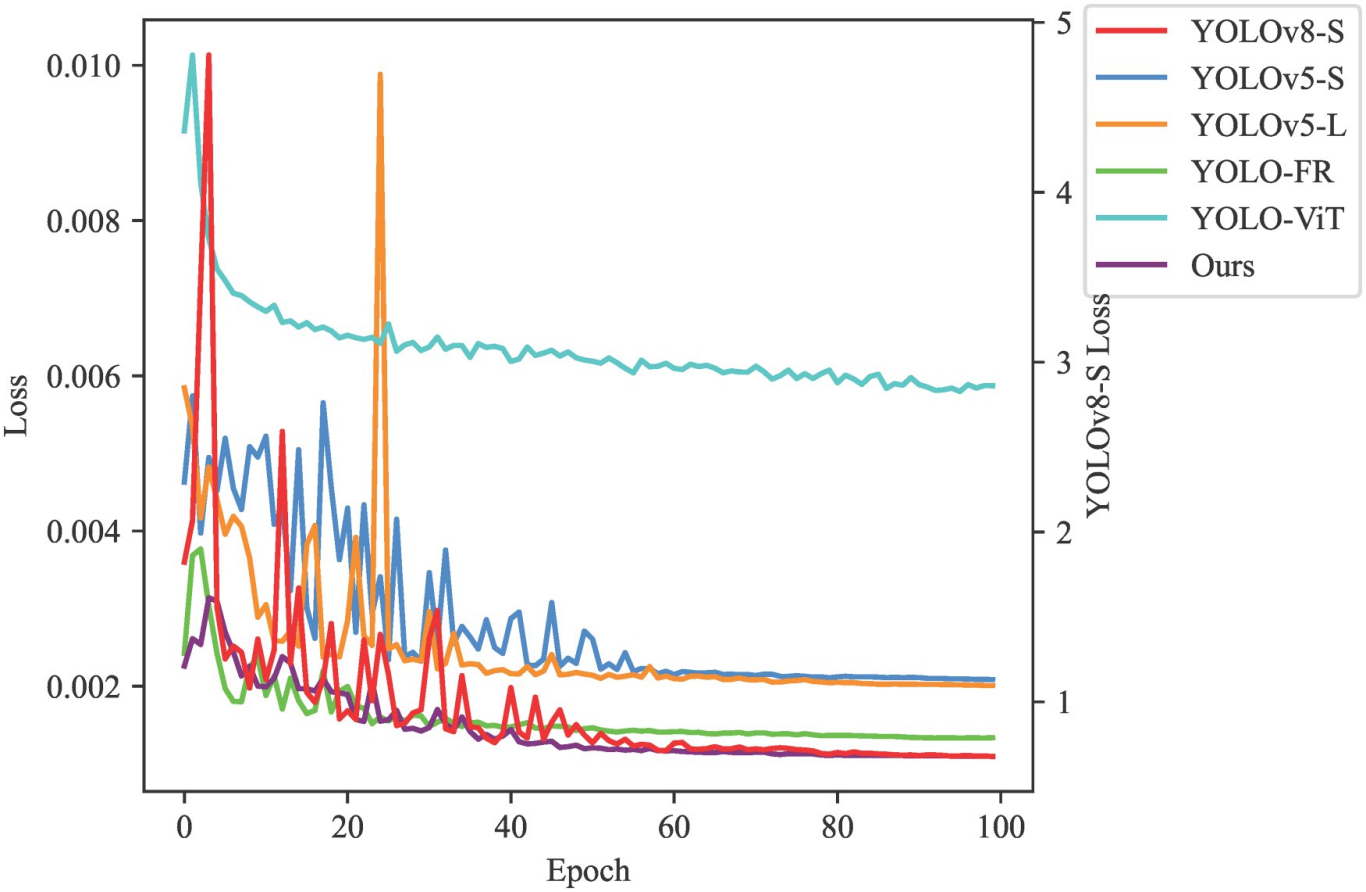
Comparison of convergence speeds of different methods on NUST-SIRST dataset.

To further validate the superiority of the improved network for infrared small target detection, we conducted several comparative experiments, including convergence speed, F1 curve, and PR curve. Firstly, we observed the variation of the loss during the training process, as shown in Fig 5. It is evident that after 40 epochs of training, the loss of YOLO-ISTD stabilizes. This indicates that our proposed YOLO-ISTD can effectively learn and adapt to the features of the data during the training process, achieving a good convergence state in a relatively short time. It can accurately predict targets and reduce prediction errors.

**Fig 6:**
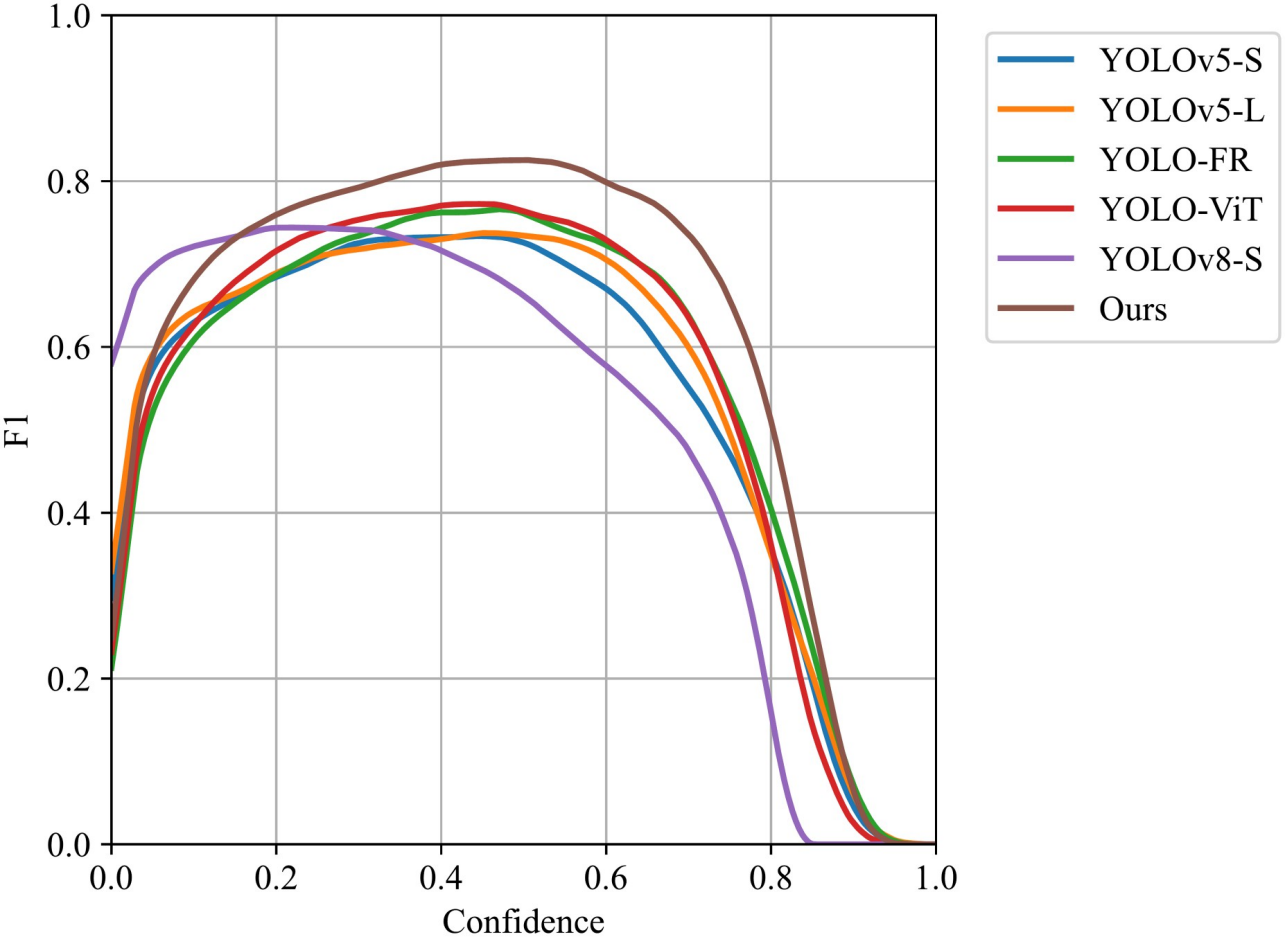
Comparison of F1 of different methods on NUST-SIRST dataset.

**Fig 7:**
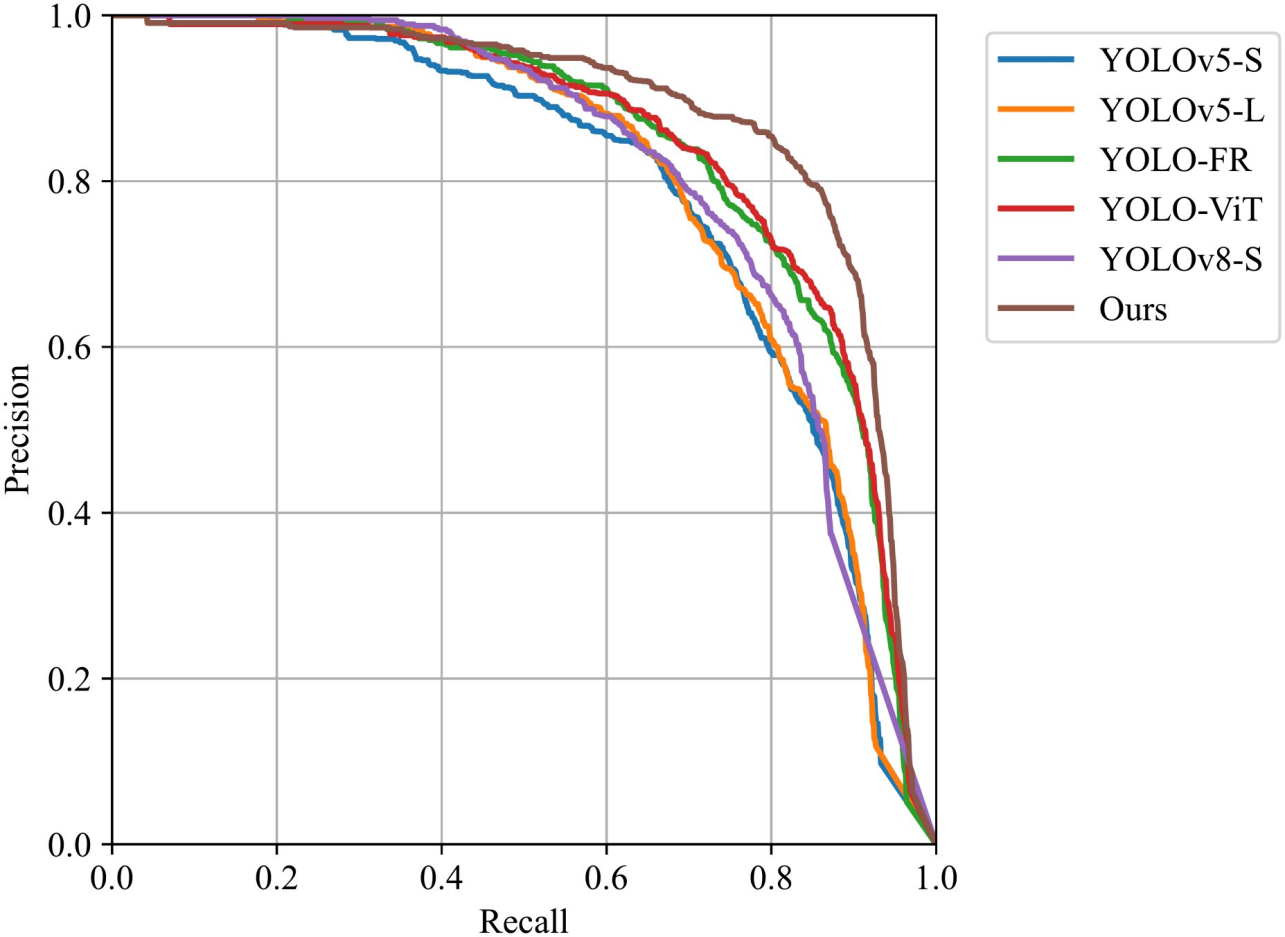
Comparison of PR of different methods on NUST-SIRST dataset.

We also evaluated the performance of the improved network by plotting Figs 6 and 7. Fig 6 shows the results of the F1 curve, while Fig 7 displays the results of the PR curve. These curves reflect the balance between target detection accuracy and recall of the improved network under different thresholds. The comparison shows that YOLO-ISTD achieves better performance in both metrics, further proving the effectiveness of our method.

In conclusion, through comparative experiments and curve analysis, our proposed YOLO-ISTD demonstrates superiority in infrared small target detection tasks. It exhibits faster convergence speed and excellent performance in terms of F1 curve and PR curve. These results provide solid data support for the reliability and practicality of our method.

**Fig 8:**
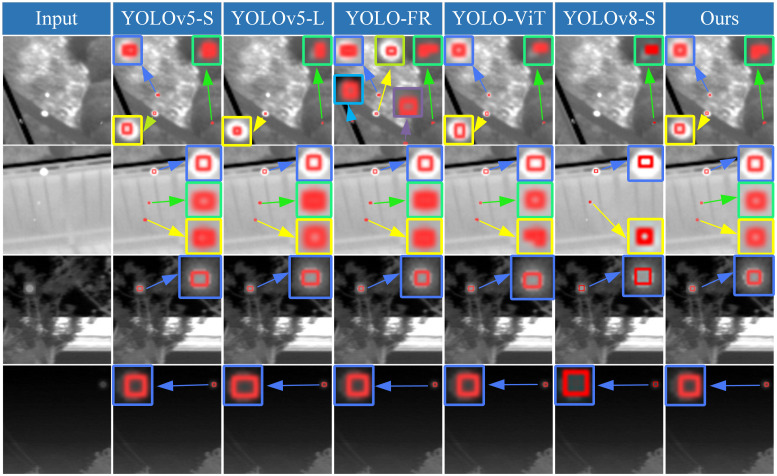
Detection results of small infrared targets in different scenes using different methods. Reprinted from [42] under a CC BY license, with permission from Wang huan, original copyright 2019.

Furthermore, based on the detection results of YOLO-ISTD on four different scenarios on the NUST-SIRST dataset shown in Fig 8, it is clear that our method has a higher level of accuracy. In these four figures, different coloured boxes indicate different targets, blue, green and yellow boxes indicate correctly predicted targets and other coloured boxes indicate false alarm targets.

### Ablation experiments

To validate the effectiveness of the YOLO-ISTD model proposed in this study, we conducted a series of ablation experiments on the NUST-SIRST dataset to assess the impact of each module on YOLO-ISTD.

Table 2 shows the results of the ablation test of YOLO-ISTD, where the detection performance of the other four improved models was improved compared to the baseline model, with the hybrid model showing the largest gain improvement, 8.568% for mAP@0.5 and 8.618% for mAP@0.95 compared to the original YOLOv5-S.

**Table 2:** Ablation test results of YOLO-ISTD.

Yolov5s	Head	NLSPPF	Anchor	SACSP	map@0.5	map@0.95
✓					78.897%	38.344%
✓	✓				83.998%	43.471%
✓	✓	✓			84.800%	43.711%
✓	✓	✓	✓		85.338%	45.037%
✓	✓	✓	✓	✓	87.465%	46.962%

**Fig 9:**
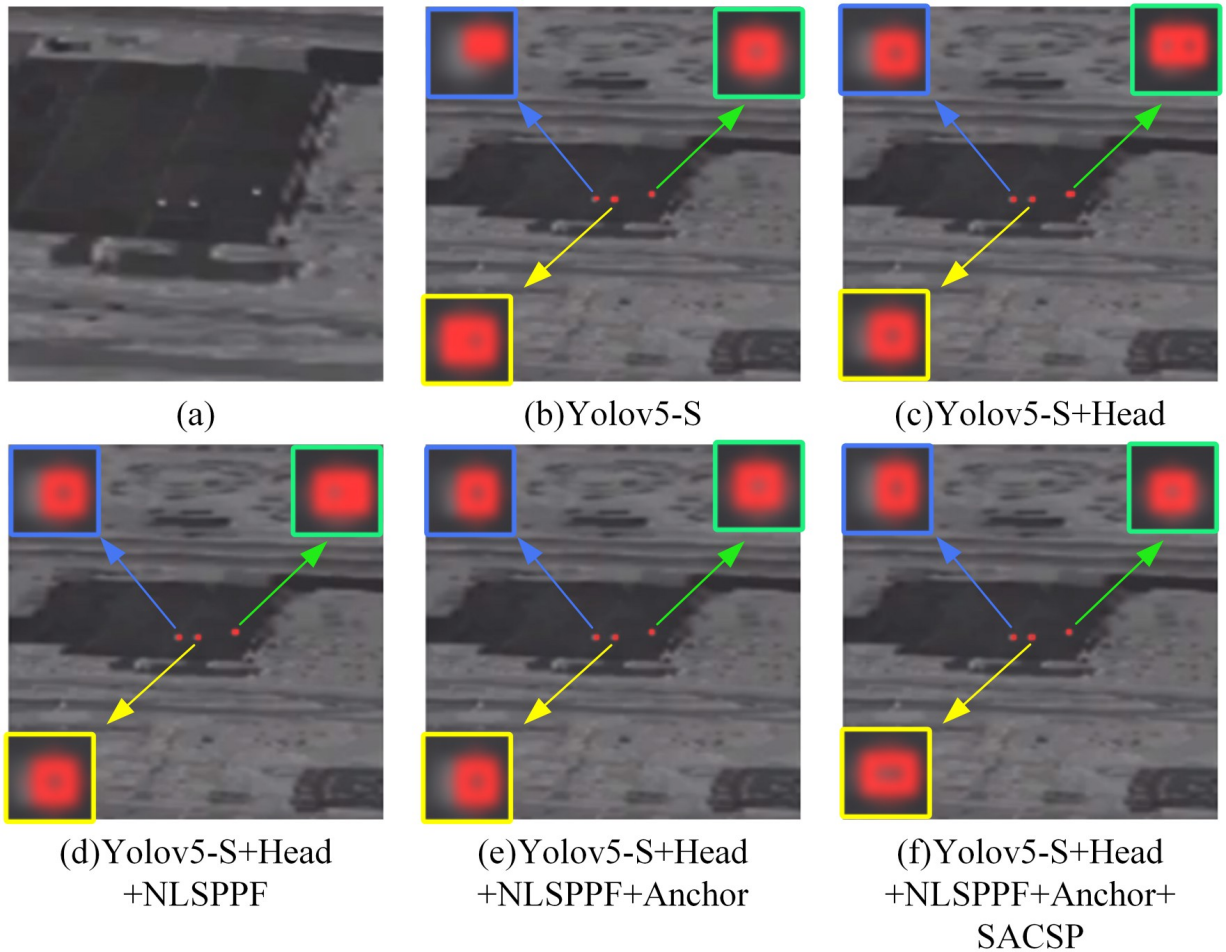
Effect of ablation experiment. Reprinted from [42] under a CC BY license, with permission from Wang huan, original copyright 2019.

The specific ablation experiment results are shown in Fig 9, the results show that they complement each other in the detection task to make the model achieve the best detection performance, and each additional improved module increases the mAP of the model.

Separate ablation experiments for each improvement step will be performed and analyzed below.

#### SACSP

To evaluate the effectiveness of the SACAP module proposed in this paper for extracting infrared small target features, we conducted ablation experiments on the NUST-SIRST dataset using the YOLO-ISTD model as the baseline model. The validation results are shown in Table 3. On mAP@0.5 and mAP@0.95, using the SACSP module of this paper improves the results by 3.778% and 2.923%, respectively.

**Table 3:** Ablation test results for CSP module.

Different CSP modules	map@0.5	map@0.95
CSP	83.687%	44.039%
SACSP	87.465%	46.962%

**Fig 10:**
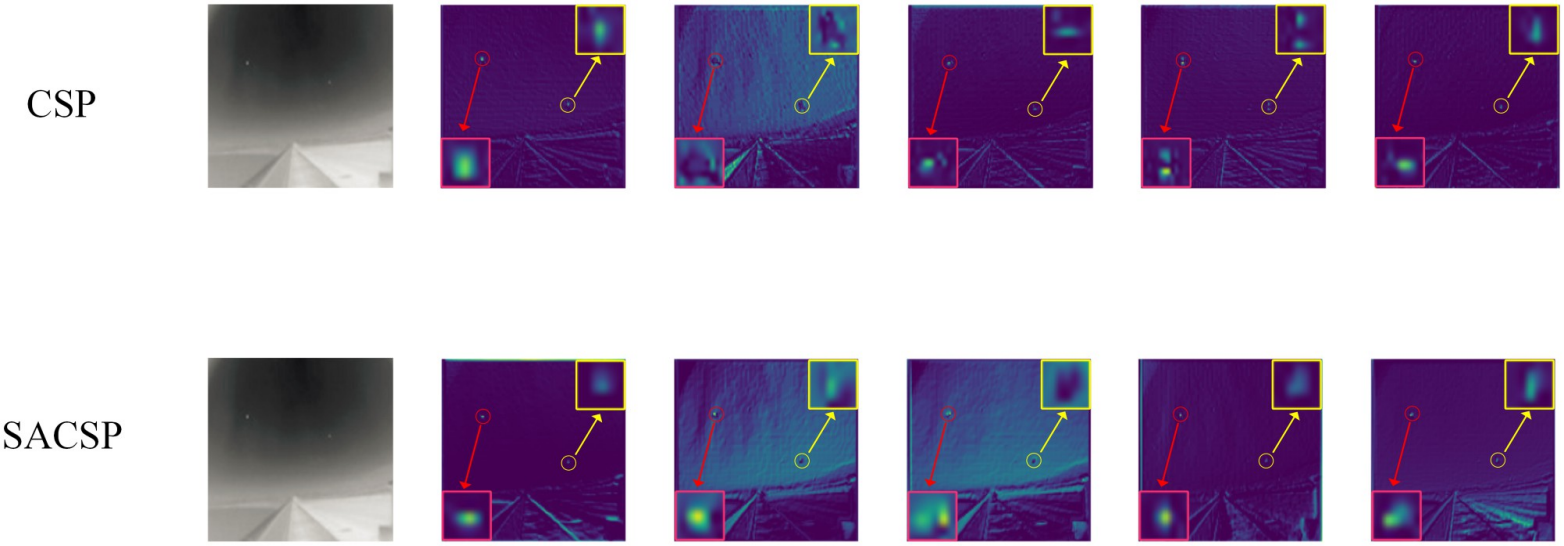
Comparison of feature maps for the output of layer 2 of the network. Reprinted from [42] under a CC BY license, with permission from Wang huan, original copyright 2019.

YOLO-ISTD outputs the shallowest feature map used for subsequent fusion in the CSP module at layer 2 of the network, which contains the most complete image features and is ideal for visual observation and comparison. In order to visually analyze the improved effect of the SACSP module on feature extraction relative to the original CSP module, we will replace Backbone’s CSP module with the SACSP module before and after the network to detect the same image and visually compare the output feature maps at layer 2 of the network. Five neighboring identical channels in the output layer 2 feature maps of the two networks were selected for observation and comparison, as shown in Fig 10.

By observing Fig 10, we can clearly see that when using the CSP module, the extracted IR small target information is mixed with the background information, which appears darker and is easy to misidentify the background information as the target. However, after using the SACSP module, the extracted small target features are much clearer and brighter, and almost completely separated from the background. This reflects that replacing Backbone’s CSP module with the SACSP module is beneficial for infrared small target detection.

#### NLSPPF

To evaluate the effectiveness of the NLSPPF module proposed in this paper for integrating multi-scale information in infrared small targets, we conducted ablation experiments on the NUST-SIRST dataset using the YOLO-ISTD model as the baseline model. The validation results are shown in Table 4

**Table 4:** Ablation test results for SPPF module.

Different SPPF modules	map@0.5	map@0.95
SPPF	86.202%	46.219%
NLSPPF	87.465%	46.962%

**Fig 11:**
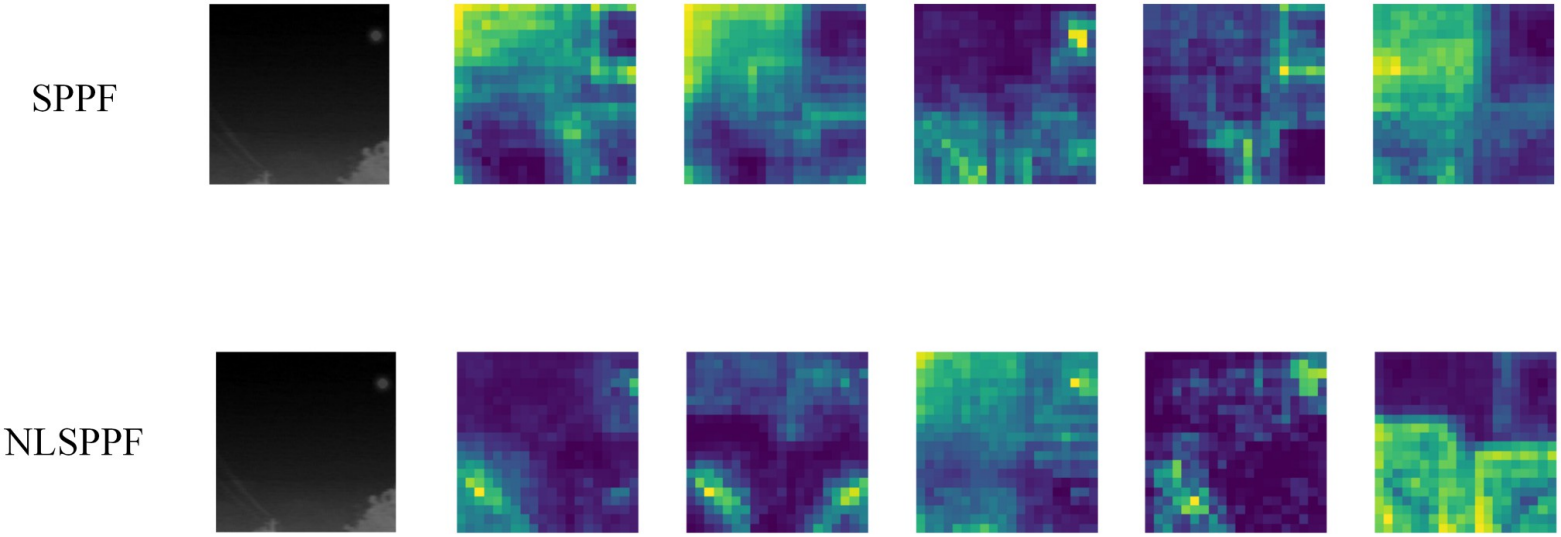
Comparison of feature maps for the output of layer 9 of the network. Reprinted from [42] under a CC BY license, with permission from Wang huan, original copyright 2019.

In order to visually analyze the improvement effect of the NLSPPF module on feature fusion relative to the original SPPF module, we will replace the networks before and after the SPPF module with the NLSPPF module for detecting the same images and visually comparing the output feature maps of the network’s layer 9. Five neighboring identical channels in the output layer 9 feature maps of the two networks were selected for observation and comparison, as shown in Fig 11.

As can be seen in Fig 11, after replacing the SPPF module with the NLSPPF module, the association calculation is performed for features in the global range. This improvement effectively suppresses the background noise and allows the model to better focus on the feature information of small IR targets. In the subsequent feature operations, the useless background information is eliminated, which improves the accuracy of the model and is more beneficial for IR small target detection.

#### Anchor

In order to explore the impact of Anchor generated by different K-means algorithms on the detection performance, comparative experiments were conducted on the NUST-SIRST dataset. Their validation results are shown in Table 5. At mAP@0.5 and mAP@0.95, the results using K-means_DIOU are 1.702% and 1.254% higher than those using K-means,respectively.

**Table 5:** Ablation test results of K-means algorithm.

Different clustering algorithms	map@0.5	map@0.95
K-means	85.763%	45.708%
K-means_DIOU	87.465%	46.962%

**Fig 12:**
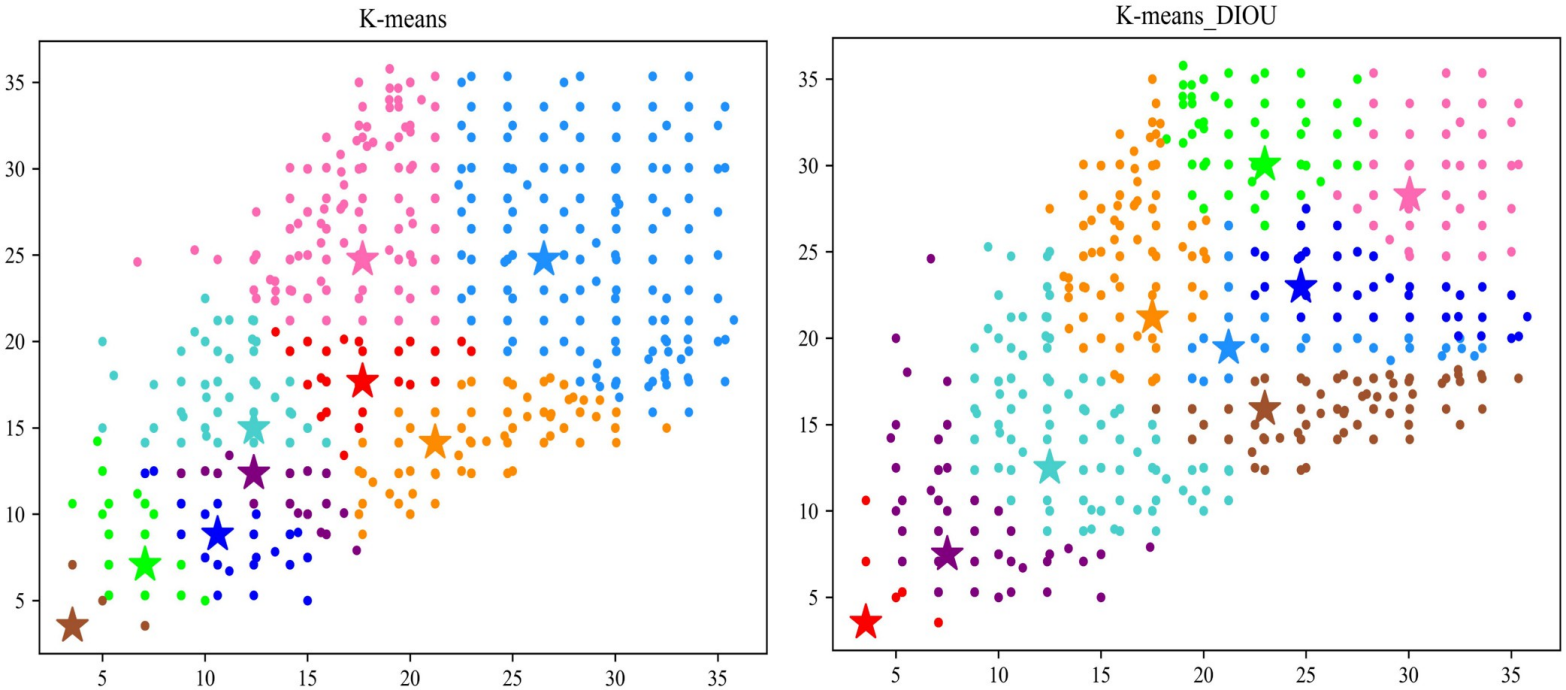
Clustering diagram of two K-means algorithms.

**Fig 13:**
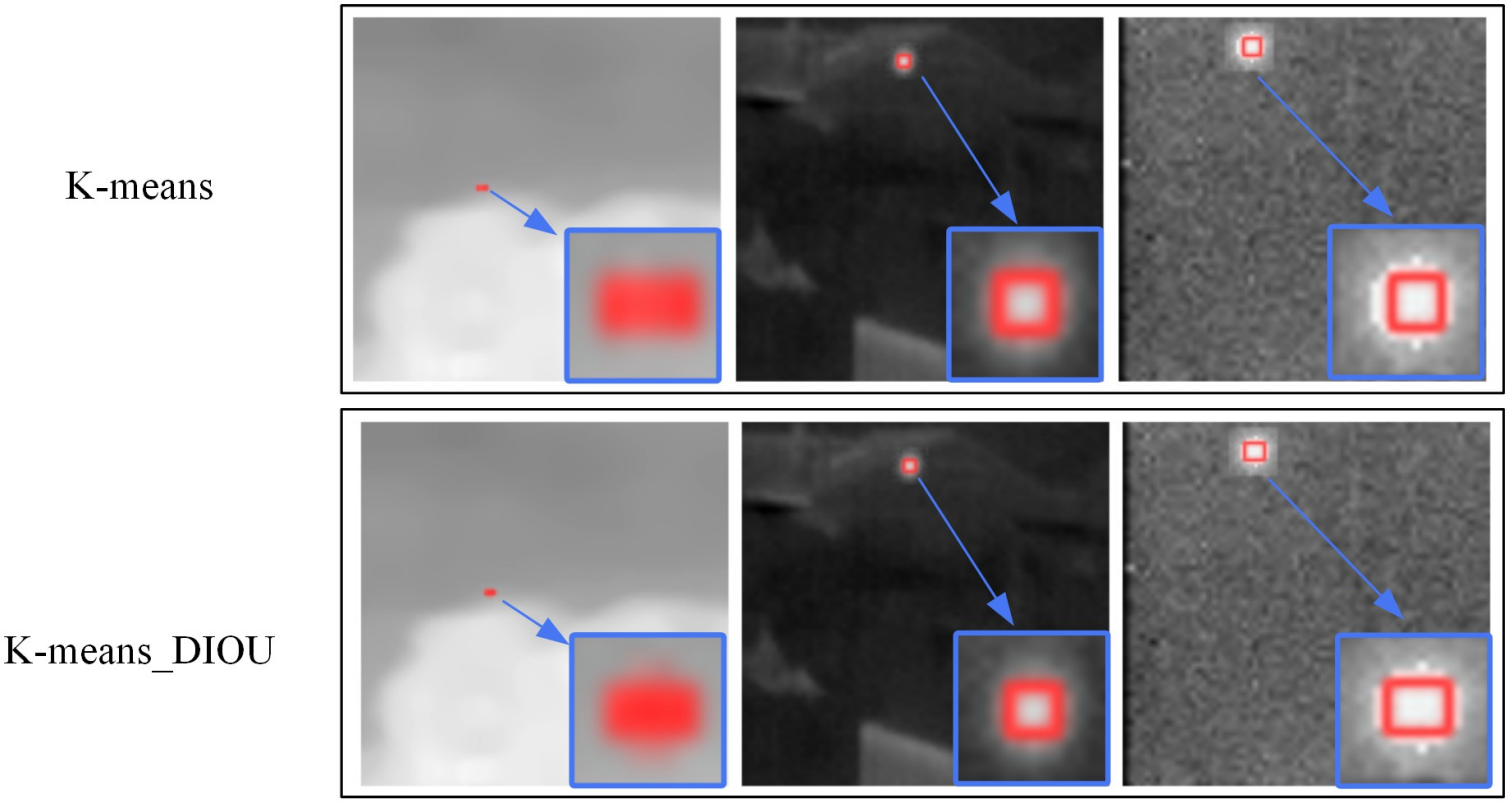
Comparison of different K-means algorithms in terms of detection performance. Reprinted from [42] under a CC BY license, with permission from Wang huan, original copyright 2019.

In addition, in order to visualise the advantages of the K-means_DIOU method over the original K-means algorithm in this paper, we show the clustering maps obtained by the two algorithms in Fig 12 and the detection maps obtained by the two algorithms separately in Fig 13.

According to the observations in Fig 12, the cluster points obtained from K-means_DIOU clustering show a more uniform distribution. This uniform distribution results in a more balanced ratio between each cluster. In the YOLO algorithm, the role of the Anchor is to determine the location and scale of possible targets. If there are too many cluster points corresponding to a particular Anchor, it may cause the model to fail to find the proper size during training. In contrast, by making the scale between each cluster more balanced, each Anchor can be made to fit as many targets as possible, thus improving the model’s adaptability. As can be seen in Fig 13, the target frame obtained with the K-means_DIOU method is closer to the target and is more effective.

## Discussion and conclusion

In this paper, an infrared small target detection method named YOLO-ISTD is proposed to solve the problems of low resolution of infrared images, small size of small targets and susceptibility to interference by making improvements in several aspects. In this method, the YOLOv5 model is used as the basic framework, and the SACSP feature extraction module is incorporated to reduce the influence of image background and noise on infrared small targets, enhancing the network’s ability to extract crucial target features and focus on them. In addition, by incorporating the NLSPPF feature fusion module, the network is able to better localize the target and improve the detection ability of small targets. And for the special characteristics of small targets in infrared target images, nine target frames that are more suitable for infrared small target detection are obtained using the K-means_DIOU clustering algorithm.

The experimental results show that by purposefully designing the deep learning model, the method achieves 87.465% of mAP@0.5 and 46.962% of mAP@0.95 on the NUST-SIRST dataset, and compared with the BaseLine model, the convergence of mAP@0.5, mAP@0.95, precision, recall and model speed are all significantly improved. In addition, compared with the SOTA method, the proposed method can better solve the problems of missed detection and false alarms in the detection results.

In addition, infrared images can exhibit significant variations under different lighting conditions, which may have a negative impact on the visibility of targets and the performance of detection algorithms. Therefore, our future plans involve focusing on exploring corresponding improvements in YOLO-ISTD for the detection of infrared small targets under different lighting conditions. Specifically, we will emphasize the optimization of feature extraction and representation methods for infrared images, aiming to adapt to target detection and recognition tasks in diverse lighting conditions and enhance the robustness of the model.
